# How Communication Technology Fosters Individual and Social Wellbeing During the Covid-19 Pandemic: Preliminary Support For a Digital Interaction Model

**DOI:** 10.1007/s10902-021-00421-1

**Published:** 2021-06-19

**Authors:** Natale Canale, Claudia Marino, Michela Lenzi, Alessio Vieno, Mark D. Griffiths, Marta Gaboardi, Matteo Giraldo, Carmen Cervone, Santinello Massimo

**Affiliations:** 1 Department of Developmental Psychology and Socialisation, University of Padova, Via Venezia 8, 35131 Padova, Italy; 2 International Gaming Research Unit, Psychology Department, Nottingham Trent University, Nottingham, UK

**Keywords:** Coronavirus-19, COVID-19, Social sharing of emotions, Online social support, Posttraumatic growth, Positive mental health, Prosocial behaviors

## Abstract

The aim of the present study was to test an explanatory model for individual and social wellbeing which incorporates the advantages of using digital technologies during the COVID-19 pandemic. The study was carried out in Italy, one of the countries that has been most severely affected by the pandemic worldwide. The study was designed to include variables that might be specifically pertinent to the uniqueness of the restrictions imposed by the pandemic. Adults living in Italy (*n* = 1412) completed an online survey during the lockdown period in March 2020. Results showed two distinct digital interaction processes highlighted by the facilitating use of online emotions (“e-motions”) and online social support (“e-support”). In short, e-motions were positively related to posttraumatic growth, which in turn was positively associated with positive mental health and higher engagement in prosocial behaviors. Moreover, individuals who perceived themselves as having greater e-support were characterized by higher levels of positive mental health, which it turn was positively associated with prosocial behaviors. Collectively, these two digital interaction processes suggest that digital technologies appear to be critical resources in helping individuals cope with difficulties raised by the COVID-19 pandemic.

## Introduction

In March 2020, Italy was the first European country to adopt measures of spatial distancing (e.g., quarantine and self-isolation; Abel & McQueen, 2020) in order to delay and mitigate the impact of the COVID-19 pandemic, and to follow the guidelines for the use of non-pharmaceutical measures proposed by the European Centre for Disease Prevention and Control (ECDC, 2020). At the time of writing, the majority of the world population has been complying with the containment regulations implemented by the respective countries. However, the American Psychological Association (APA) recently stated that spending entire weeks at home (with limited resources, stimulation, and social contacts) can seriously damage individuals’ health and wellbeing, by increasing negative internal states, such as fear, anxiety, depression, frustration and/or irritability (APA, 2020) as has also been demonstrated by recent studies specifically examining the psychological impact of COVID-19 both in Italy (Cellini et al., 2020; Soraci et al., 2020) and other countries (e.g., Ahorsu et al., 2020; Sakib et al., 2020). Moreover, there are expectations about significant escalations in mental health problems among the general population after the current pandemic crisis ends (Galea et al., 2020), especially in countries characterized by economic vulnerability and negative economic consequences such as Italy and Spain (Codagnone et al., 2020).

Despite such difficulties, individuals have also reacted by engaging in altruistic behaviors, such as volunteering, donating money, and offering online social and emotional support to others (Aresi et al., 2020; Brooks et al., 2020). Unique to COVID-19 is the widespread access to digital communication technologies that helps buffer the negative outcomes related to the COVID-19 pandemic, for example by reducing loneliness and isolation and increasing belongingness via social support (Gabbiadini et al., 2020). Although the recent literature has already studied the negative psychological impact of COVID-19 (see Salari et al., 2020 for a meta-analysis), few studies have focused on how positive outcomes may occur despite adversities. Therefore, the present study examined the specifics of how digital communication technologies can help individuals coping with the difficulties raised by home confinement through fostering individual and social wellbeing.

Because of the restrictions imposed during the ongoing pandemic, individuals were not allowed to use common coping strategies to manage the difficult conditions of quarantine and isolation, such as going to the gym, attending sporting events, going to the cinema/theatre, or attending religious services. As an alternative, a useful coping strategy is staying *virtually connected with others* because virtual conversations (e.g., phone calls, text messages, video chats, and interaction on social media) can guarantee access to social support networks and allow individuals the opportunity to discuss their own experiences and associated emotions (APA, 2020). Over the past two decades, digital communication technologies (i.e., online support groups, blogs, social media) have been found to positively influence psychological outcomes in mass disaster contexts, such as Hurricane Katrina, terrorist attacks, and the earthquake in Haiti (e.g., Brunet et al., 2010). Two main social interaction processes have been highlighted following disastrous events: (i) online sharing of emotions (Rimé et al., 2020) and (ii) online social support (Herbert & Brunet, 2010).

## Sharing Emotions Online

In exceptional circumstances, such as natural disasters (e.g., hurricanes and earthquakes), traumas are experienced by entire communities bringing out collective traumatic dynamics (Lowe et al., 2013; Manove et al., 2019; Wlodarczyk et al., 2016). Taking into account the health and social impact of COVID-19 among the Italian population, the ongoing COVID-19 pandemic presents characteristics of collective trauma experiences that have stimulated national identity and interpersonal trust (Ellena et al., 2020). Collective traumas have been found to elicit intense sharing of emotions (offline and online) among members of concerned communities (Rimé et al., 2020). Considering the worldwide increased usage of online digital technology during the coronavirus lockdown (Perez, 2020), it is plausible that individuals have shared their emotions using digital technologies, such as smartphones and social media. In fact, a recent study found that the COVID-19 outbreak has resulted in excessive emotional experiences in users of *Weibo* (a leading Chinese online social networking platform) (Li et al., 2020).

In the context of the widespread tendency to express and manage emotions in the cyberspace, Zych and colleagues (2017) introduced the multidimensional concept of “e-motions”, that is the expression, perception, use, and regulation of emotions online. Drawing from the emotional intelligence literature (Mayer & Salovey, 1997), e-motions comprise four main factors including (i) emotional expression and (ii) emotional perception on social media, (iii) understanding and management of emotions online, and (iv) beliefs about the facilitating role of emotion-expression on social media in overcoming personal difficulties (Marino et al., 2020; Zych et al., 2017). The latter (facilitating use of online emotions, henceforth “e-motions”) was chosen for the purpose of the present study because the belief that the expression of e-motions may improve relationships and help in thinking as well as making decisions might be particularly relevant in pandemic times characterized by (inter)personal difficulties.

Online emotional disclosure elicits emotional responses (Bareket-Bojmel & Shahar, 2011), emotional support (Pentina, & Zhang, 2017), and reinforces involvement in online social behaviors (Erreygers et al., 2017). Online sharing of emotions also has clear implications in the processes of mourning and reaction to traumatic events (Neubaum et al., 2014; Tedeschi, & Calhoun, 2004) by fostering the reconstruction and reorganization of positive socially shared beliefs about the self and the world. Emotions are evolutionary adaptations that guide individuals in responding to environmental challenges (Nesse & Ellsworth, 2009). Mixed emotions have been found to be associated with eudaimonic wellbeing (e.g., person’s sense that his or her life has purpose or meaning; Berrios et al., 2018; Fredrickson, 2013). Higher sharing of emotions after a disaster (e.g., March 2004 terrorist attack in Madrid and November 2015 terrorist attacks in Paris) predicted higher social integration, posttraumatic growth, and prosocial behavior (Garcia, & Rimé, 2019; Rimé et al., 2010).

Several authors have argued that exposure to the COVID-19 pandemic and its related consequences can also be considered an (individual) traumatic event with almost 20% of adults among the general population suffering posttraumatic stress disorder symptoms during the early stages of the current pandemic (Karatzias et al., 2020; Vasquez et al., 2021). Vasquez et al. (2021) argued that posttraumatic growth, just after the onset of the pandemic crisis, could be considered an initial coping strategy that allows the development of long-term positive outcomes in personality. Posttraumatic growth has been defined in the field of Positive Psychology as the experience of positive changes that follows highly challenging life crises (e.g., life-threatening diseases, Tedeschi & Calhoun, 2004), which also includes the human capacity for resilience (e.g., “the ability to maintain a stable equilibrium”; Bonanno, 2004, p. 20) and the use by individuals of aversive events as a springboard for further growth and development (Bonanno, 2004). It is manifested by increased appreciation of life and sense of personal strength, changed priorities, a richer existential and spiritual life, and more meaningful interpersonal relationships, which result in greater subjective wellbeing (e.g., Arpawong et al., 2016; Dickinson, 2020; Ryff & Singer, 2008). In addition to the intrapersonal benefits of posttraumatic growth, other studies have demonstrated prosocial behaviors following trauma as a form of posttraumatic growth (e.g., Frazier et al., 2013). From this viewpoint, in a situation characterized by increased depression and isolation, it is extremely important to adopt a strength-based approach in the context of emergencies and traumatic experience, in order to nurture individuals’ capacity to grow from adversity (Dickinson, 2020).

## Online Social Support

Social support can be considered a psychosocial protective factor in relation to mental health difficulties during the current pandemic. Perceived social support relates to the subjective evaluation of how individuals perceive someone (e.g., friends and family members) as being available to provide material, psychological, and overall support during times of need (Eagle et al., 2019). In non-pandemic situations, several studies have suggested that social support is crucial for individual wellbeing (Cohen, 2004; Leigh-Hunt et al., 2017; Siedlecki et al., 2014). A large body of research has shown that social support is positively associated with mental health in terms of positive affect, self-esteem (e.g., Cohen & Wills, 1985), and adjustment after a negative experience (e.g., Forbes & Roger, 1999). A recent review of findings in the area of disaster mental health confirmed that survivors’ perception of being supported is essential for survivors’ wellbeing and, in turn, improves the quality of interpersonal relationships, for example in terms of intentions to help others and general prosocial behaviors (Kaniasty, 2020).

Social support also helps individuals by alleviating posttraumatic reactions (e.g., Ozer et al., 2003) and by promoting posttraumatic growth (e.g., Jia et al., 2015; Yu et al., 2014), for example, by bolstering the ability to cope with imposed demands. Several recent studies already demonstrated that higher levels of perceived social support mitigate the effect of spatial distancing mandates during the COVID-19 pandemic by reducing loneliness, depression, and PTSD, and increasing belongingness (e.g., Gabbiadini et al., 2020; Grey et al., 2020; Li et al., 2021).

During the COVID-19 pandemic, individuals’ feelings of connectedness toward their social relationships dramatically changed due to stay-at-home mandates and limited social contact because individuals were asked to work from home, keep distance from others, and engage in self-quarantine (World Health Organization, 2020). In this scenario, online technologies for communication have functioned as a social connector by maintaining social connections and facilitating the exchange of online social support among individuals living in home confinement (Gabbiadini et al., 2020). Online social support concerns the sense of identity and belonging experienced by individuals when they feel understood and respected during a process of emotional, information, and material exchange in online interpersonal interactions. Online social support refers to an expansion of social support from real life to cyberspace, which can improve the physical and mental health of individuals (Gilmour et al., 2020) and can be provided at any time and shared beyond geographic boundaries. Also, digital technologies provide opportunities to give and receive social support to individuals, especially for those who are experiencing isolation at home. In the context of the COVID-19 pandemic online social support (henceforth “e-support”) represents the main form of social support many individuals have access to, and is therefore an important issue in the field of cyberpsychology (Guitton, 2020).

## The Present Study

During the COVID-19 pandemic, it is imperative to understand how the population, especially that of severely affected countries such as Italy, has been coping with such difficult conditions of quarantine and isolation. Some recent studies have reported the psychiatric impact of the novel coronavirus outbreak (see Brooks et al., 2020 for rapid review of the evidence). However, few studies have reported the impact of strategies to cope with the impact of COVID-19 pandemic on positive mental health or quality of life (e.g., Zhang & Ma, 2020). The uniqueness of the restrictions imposed by the pandemic appear to be an unprecedented research opportunity to test models that take into account the advantages of digital technologies for communications, virtual meeting, and expression of emotions and feelings.

**Fig. 1:**
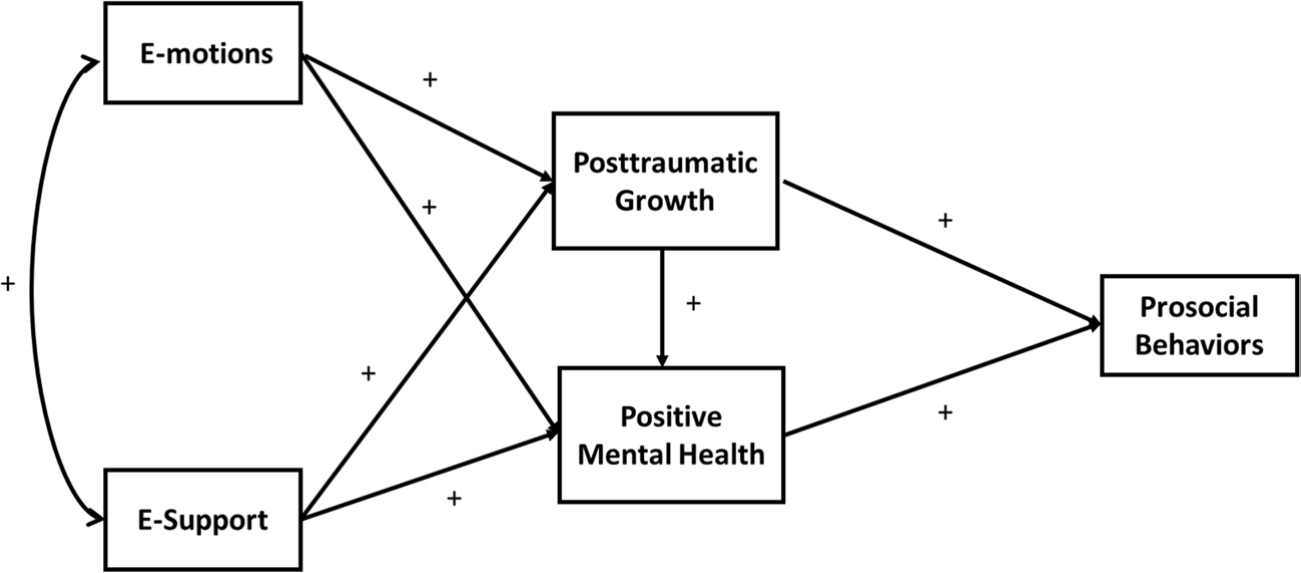
The proposed digital interaction model

Consistent with the reviewed theoretical backgrounds, the present authors developed the digital interaction model for individuals living the COVID-19 pandemic, depicted in Fig. 1. The top half of the figure suggests that e-motions lead to posttraumatic growth and positive mental health, which in turn are positively associated with prosocial behaviors (H1). The bottom half of the figure posits that perceived e-support leads to posttraumatic growth and positive mental health, which in turn are related to prosocial behaviors (H2). For the e-motions path to prosocial behaviors, it was hypothesized that there would be indirect effects mediated by posttraumatic growth and positive mental health (H3). For the e-support path to prosocial behaviors, it was hypothesized that there would be indirect effects mediated by positive mental health and posttraumatic growth (H4). Also, considering that previous studies found a positive relationship between social support and self-disclosure in general (Lee et al., 2013; Liu & Brown, 2014), it was hypothesized there would be a positive association between e-support and e-motions (H5). Taken together, the two digital interaction processes (e-motions and e-support) can make digital interactions critical resources in helping individuals cope with difficulties raised by the COVID-19 pandemic.

## Methods

### Procedure and Participants

Participants in the present study were recruited via an online survey powered by Qualtrics® during the lockdown period in March 24–31 2020 (third week of the Italian lockdown). The lockdown was decreed by the Italian government due to the emergency caused by COVID-19. All participants received information about the study and gave their online consent before starting the survey. The anonymity of the participants was guaranteed (no personal data or Internet Protocol address was collected). No compensation was given for participating in the study. The research team’s university ethical committee provided approval for the study (protocol number: 3540). The study did not involve human and/or animal experimentation and conformed to all guidelines according to the Declaration of Helsinki. A total of 1413 participants completed the survey. From this sample, one participant was excluded because he reported having been infected by COVID-19. The final sample consisted of 1412 adults living in the Italian territory (415 males; M_age_ = 36.68 years (SD ± 14.43), age range = 18–86). Regarding their education, the majority (59%) had a higher education level (Bachelor's or Master's degree or PhD), 35% had a high-school diploma, and the remaining 5% comprised elementary, secondary, or other. Regarding cohabitation status during the lockdown, 11% reported living alone, 25% reported living with one other individual, 24% reported living with two other individuals, 25% reported living with three other individuals, 10% reported living with four other individuals, and 4% reported living with five or more individuals. The study was part of a larger research project on the psychosocial impact of the COVID-19 pandemic in Italy, and other data not related to the present study will be presented elsewhere.

### Measures

Psychometric scales included in the online survey were selected to prioritize instruments that had been validated and published in Italian. The scales were presented in random order to control for the influence that the order of presentation of different constructs might have had on answers. For the present study, participants were asked to answer to each scale thinking specifically about the current ongoing period of the COVID-19 emergency in Italy.

*Online social support* was assessed using the Italian version of the Offline/Online Social-Support Scale (Wang & Wang, 2013, adapted from Leung & Lee, 2005; Italian version: Mazzoni et al., 2016). The online social support subscale comprises 11 items (e.g., “someone you can count on to listen to you when you need to talk”) rated on a four-point scale from 1 (never) to 4 (often). Participants were asked to rate the frequency with which they have other individuals available to fulfill emotional and social needs. Higher scores indicate a higher level of perceived online support. Cronbach's alpha in the present study was 0.96 (95% CI [0.95, 0.96]).

*Facilitating use of e-motions* was assessed by using one dimension of the E-motions Questionnaire (Zych et al., 2017; Italian version: Marino et al., 2020). The dimension comprises four items rated on a five-point scale from 1 (completely disagree) to 5 (completely agree). Participants were asked to rate their agreement with each item thinking about their use of social media to facilitate emotion expression, thoughts, and relationships with others during the specific situation of the COVID-19 pandemic. Therefore, items were slightly adapted to the emergency context (e.g., “I express my emotions on social networks to overcome my difficulties *in this situation*”). Higher scores refer to greater facility in expression e-motions. Cronbach's alpha in the present study was 0.80 (95% CI [0.78, 0.82]).

*Posttraumatic growth* was assessed using the Short Form of the Posttraumatic Growth Inventory (Italian version: Prati & Pietrantoni, 2014). It comprises ten items (e.g., “I discovered that I’m stronger than I thought I was”) evaluating positive changes after a traumatic experience. The PTGI is scored with a six-point Likert-type scale ranging from 1 (no change) to 6 (high change), with higher scores referring to greater posttraumatic growth. Cronbach's alpha in the present study was 0.91 (95% CI [0.90, 0.92]).

*Mental wellbeing* was assessed using the Warwick-Edinburgh Mental Well-Being Scale (WEMWBS; Italian version: Gremigni & Stewart-Brown, 2011). This scale encompasses most aspects of subjective wellbeing (hedonic perspective) and psychological functioning (eudaimonic perspective) addressing aspects of positive mental health. The scale consists of 14 positively worded items (e.g., “I've been feeling relaxed, I have been thinking clearly”), each rated on a five-point scale from 1 (never) to 5 (always). Higher scores reflect a higher level of mental well-being. Cronbach's alpha in the present study was 0.86 (95% CI [0.85, 0.87]).

*Prosocial behavior* was assessed with three items asking participants how often they had been involved in different prosocial activities during these times of emergency. More specifically: (i) volunteering in activities for the elderly and individuals in need; (ii) donating to services that are working to contain the sanitary/social emergency caused by COVID-19; (iii) helping individuals in other ways. Items were adapted from Caprara et al. (2005) by selecting the most relevant activities for the current emergency and combining volunteering, informal help, and donations. Responses ranged from 1 (never) to 5 (all the time). Due to the fact that this measure consists of only three items and the type of Likert scale was discrete and ordinal, the factorial properties were evaluated with polychoric correlations between item: volunteering and donations (r_Polychoric_ = 0.23, CI low = 0.16—CI high = 0.30, *p* < 0.001), volunteering and help (r_Polychoric_ = 0.32, CI low = 0.26—CI high = 0.39, *p* < 0.001) and donations and help (r_Polychoric_ = 0.18, CI low = 0.12—CI high = 0.24, *p* < 0.001).

### Statistical Analyses

Descriptive statistics were computed for all the variables in the study, and bivariate Pearson’s correlations were performed. The Lavaan package (Rosseel, 2012) of the open-source software R (R Development Core Team, 2013) was used to compute the model and estimate parameters. The pattern of associations specified by the proposed model was analyzed via path analysis by using a single observed score for each variable examined in the model. The dimensions of e-motions and e-support were included as exogenous variables (independent variables; IVs), posttraumatic growth and positive mental health (mediators), and prosocial behaviors (dependent variable; DV) as endogenous variables. Relevant socio-demographic variables (age, gender, cohabiting status, and education) were included in the model as covariates of prosocial behaviors. Standardized parameters were estimated by using the robust maximum likelihood methods (MLR; Satorra & Bentler, 1994) suitable for non-normally distributed variables. In the final model, indirect paths from IVs to DV via mediators were tested using Sobel tests for mediation (Baron & Kenny, 1986; Hayes, 2013). To evaluate the overall goodness of fit of the model, the R^2^ of each dependent variable and the total variance explained by the model were considered [total coefficient of determination (TCD); Jöreskog & Sörbom, 1996]. Due to the large sample size, the level of significance was set at *p* < 0.01.

## Results

Table 1 summarizes the means, standard deviations (SDs), skewness, kurtosis, and bivariate correlations among the study variables. E-motions showed a non-normal distribution, further supporting the use of the MLR estimator for the subsequent path analysis. Overall, the sample reported a mean score of 1.74 in facilitating use of e-motions. This score was close to the second point of the five-point scale, indicating a mild agreement concerning the use of emotions to facilitate thought and relationships with others. Regarding the Online Social Support Scale, the mean score of the sample (2.74) was very close to the third point of the four-point scale, suggesting a strong agreement of perceived online support. The PTGI mean score of the sample (2.87) was close to the third of the six-point scale, indicating mild positive changes after a traumatic experience. Concerning the WEMWBS, sample’s mean score (3.35) was between the third and fourth points of the five-point scale. This score suggested a relatively high level of mental wellbeing during the COVID-19 pandemic. Finally, regarding the assessment of prosocial behaviors during the COVID-19 pandemic, the sample showed a mean score of 2.09, indicating a small involvement in prosocial activities, such as volunteering, informal help, and donations. The magnitude of correlation coefficients was relatively modest, ranging from 0.06 to 0.29.

**Table 1:** Mean (M), standard deviations (SDs), skewness, kurtosis and correlation between variables

	Mean	SD	Range	skewness	kurtosis	1	2	3	4	5
1.E-motions	1.74	0.02	1–5	1.33	1.91	–				
2.E-support	2.74	0.02	1–4	−0.08	−0.83	0.13***	–			
3.Posttraumatic growth	2.87	0.03	1–6	0.17	−0.88	0.25***	0.10***	–		
4.Positive mental health	3.35	0.91	1–5	−0.37	0.47	0.06	0.18***	0.28***	–	
5.Prosocial behaviors	2.09	0.02	1–5	0.37	−0.43	0.13***	0.08**	0.29***	0.27***	–

*N* = 1412; **p < .01; *** *p* < .001

(Cohen, 1988

**Fig. 2:**
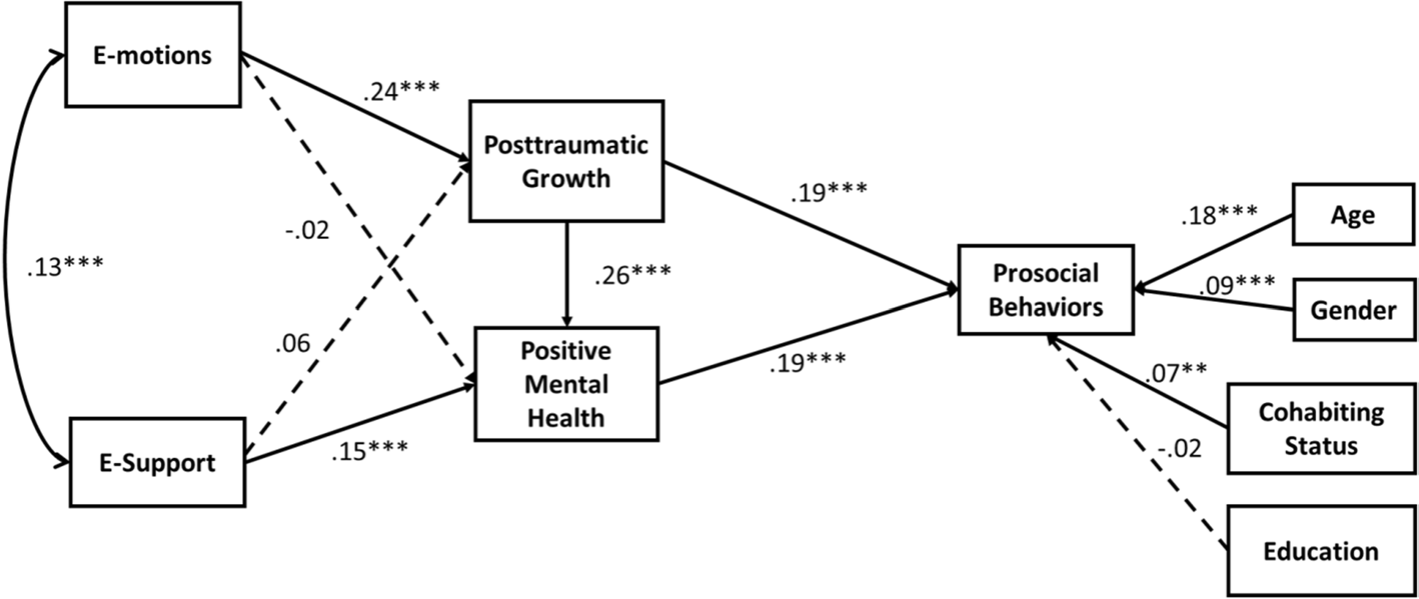
Standardized parameters for the model. *Note: N* = 1412; (**p < .01; *** p < *.001); Gender: 1* = *M; 2* = *F; Cohabiting Status: 0* = *alone; 1* = *not alone; Education: 0* = *low; 1* = *high*

Estimation of the model (Fig. 2) showed that the total coefficient of determination (TCD) was 0.13 [corresponding to a correlation of *r* = 0.36 which is a small-to-medium effect size according to Cohen’s traditional criteria, (Cohen, 1988)], and the squared multiple correlations indicated a modest proportion of the variance explained for study variables (7% in posttraumatic growth, 9% in positive mental health, and 13% in prosocial behaviors). Individuals who expressed more e-motions were more likely to report greater posttraumatic growth, which in turn was associated with a higher frequency of prosocial behaviors. A distinct pathway was observed for higher levels of e-support. Participants perceiving more e-support had higher positive mental health, which in turn was associated with a higher frequency of prosocial behaviors.

Moreover, higher posttraumatic growth was associated with higher positive mental health and a positive association was found between e-motions and e-support. Results of the Sobel tests supported the mediating role of posttraumatic growth in the association between e-motions and prosocial behaviors (β = 0.05, SE = 0.009, z = 5.16, *p* < 0.001) and positive mental health in the association between e-support and prosocial behaviors (β = 0.03, SE = 0.007, z = 4.30, *p* < 0.001). Finally, e-motions were not significantly related to positive mental health, as well as e-support to posttraumatic growth. Given these results, the mediation effects of positive mental health in e-motions/prosocial behaviors and posttraumatic growth in e-support/prosocial behaviors are not presented.

## Discussion

The aim of the present study was to test an explanatory model for individual and social wellbeing which utilizes the advantages of using digital technologies during the COVID-19 outbreak in the Italian population, one of the most severely affected countries in the world (WHO, 2020). The study was designed to include variables that might be specifically pertinent to the uniqueness of the restrictions imposed by the pandemic scenario and to the current life-threating pandemic. The results of this preliminary cross-sectional study provide a relevant and original contribution concerning the distinct role of the facilitating use of e-motions and receiving e-support for psychological wellbeing and prosocial behaviors in this new worldwide situation (home confinement and spatial distancing due to COVID-19). In doing so, the present study provides empirical evidence that using technology for online interactions is an effective response to the COVID-19 pandemic for bolstering subjective wellbeing and positive mental health, as theoretically suggested by Bavel et al. (2020). A discussion of the more specific findings is highlighted below.

Regarding the online sharing of emotions, the resulting model suggested that e-motions were positively associated with posttraumatic growth, which in turn was positively associated with better mental health and higher engagement in prosocial behaviors. The results also confirm the important mediating role of posttraumatic growth in the relationship between e-motions and prosocial behaviors. These results are reminiscent of studies concerning the psychological benefits of social media use for individuals experiencing disaster situations (Neubaum et al., 2014; Nilsen et al., 2018) showing that individuals who more actively engaged in social media communication felt emotionally relieved as a part of a like-minded community. Consequently, social media applications might serve as spaces for social sharing of emotions to exchange feelings and critical thoughts about the COVID-19 situation.

It is possible that the sharing of emotions can contribute to the reconstruction and reorganization of positive socially shared beliefs about the self and the world, thus fulfilling psychological needs (e.g., needs for positive meaning, needs for collective and personal self-esteem enhancement) (Epstein, 1993) and emphasizing collective positive reactions such as altruistic and prosocial behaviors (Janoff-Bulman, 2004; Rime et al., 2010). Indeed, posttraumatic growth is generally characterized by a stronger appreciation for life, increased personal strength, and more positive relationships with others. As shown by past research, this can translate to positive personal changes (e.g., engaging in health-promoting behaviors) as well as positive interpersonal outcomes, such as a higher willingness to help others and the tendency to behave in a more altruistic way (El-Gabalawy et al., 2020; Shakespeare-Finch & Barrington, 2012).

The facilitating use of e-motions may help in coping positively with negative events, therefore turning traumatic experiences into an opportunity to grow from adversity. Use of e-motions might play multiple roles in the process of growth during the COVID-19 pandemic and may contribute to posttraumatic growth by alleviating intrusive rumination (Lepore et al., 2000) and fostering more reflective and deliberate rumination (Treynor et al., 2003) and a greater sense of intimacy and closeness (Tedeschi & Calhoun, 1996).

Previous studies have reported positive associations between openness to experience, emotional expression, and posttraumatic growth (e.g., Jaarsma et al., 2006). It is possible that e-motions lead to posttraumatic growth by fostering positive core beliefs (e.g., openness to the future and identification with humanity; Vasquez et al., 2021; Ellena et al., 2020) and emotional strengths (e.g. bravery, humor and social intelligence, Casali et al., 2021). Beliefs about the facilitating expression of e-motions for overcoming difficulties might also reflect a more general phenomenon of digital emotion regulation. According to Wadley and colleagues (2020), digital emotion regulation refers to the use of digital technologies to strategically influence affective states (including emotions, moods, and stress levels). Therefore, it is possible that at least some digital usage during the COVID-19 pandemic will be involved in the engagement in positive psychological strategies (e.g., as a means of coping with stressful situations by thinking, of evoking desired emotions and/or of increasing arousal).

Contrary to the hypotheses, e-motions were not associated with positive mental health and analysis did not confirm the mediating role of positive mental health in the relationship between e-motions and prosocial behaviors. These findings are consistent with previous studies showing that emotional disclosure improves posttraumatic growth but does not produce greater improvement in psychological symptoms (i.e., Slavin-Spenny et al., 2011; Smyth et al., 2008). It is plausible that e-motions might foster changes in beliefs and perspectives (i.e., growth) soon after the onset of the crisis, but simultaneously counter or prevent an individual from feeling better when the stressful situation (the COVID-19 pandemic) is still present (Vazquez et al., 2021).

Regarding receiving online social support, findings indicated that individuals who perceived more online social support during the COVID-19 pandemic were characterized by higher levels of positive mental health, which it turn was positively associated with prosocial behaviors. Receiving online social support via social media and other online platforms provides immediate and long-term relief in terms of sense of belonging and reduction of feelings of loneliness and isolation from others, which result in improved wellbeing and mental health (Frison & Eggermont, 2016; Herbert & Brunet, 2010). The findings are also consistent with previous studies showing that intentions to help others and general prosocial behaviors are more frequent among individuals with higher psychological wellbeing (e.g., Layousa et al., 2017; Lyubomirsky et al., 2005).

It is possible that e-support leads to positive mental health and prosocial behaviors (through positive mental health) by fostering the satisfaction of basic psychological needs, as recently found in a study among the Italian population (Smorti et al., 2021). Previous studies have indeed shown that receiving social support helps individuals feel (i) more confident in their skills (need for competence), (ii) more connected with those around them (need for relatedness), and (iii) like more willful agents of their choices (need for autonomy) (Cho et al., 2020; Tian et al., 2016), also during the COVID-19 pandemic (Zhou & Yao, 2020). As shown in several studies (see Diener et al., 2017 for a review), subjective wellbeing is associated with a variety of positive outcomes, including prosocial behavior. Indeed, positive mental health can make individuals more grateful and more aware of other individuals’ needs, consequently fostering the skills and motivation to be more prosocial. Therefore, considering the restrictions implemented by governments because of the COVID-19 pandemic, having the opportunity to benefit from online forms of social support through calls, video meetings, social media, and messages may be particularly important to stem negative consequences, maintain psychological proximity, and promote prosocial behaviors.

Contrary to the hypotheses, e-support was not associated with posttraumatic growth and analysis did not confirm the mediating role of posttraumatic growth in the relationship between e-support and prosocial behaviors. Previous research has found that social support influences trauma victims’ cognitions, including appraisals about the cause of the trauma, attributions of self-blame, and perceived dangerousness of the world (Belsher et al., 2011; Robinaugh et al., 2011). However, Woodward et al. (2015) found that support from friends and family was negatively associated with posttraumatic cognitions while no relationship was found between support from a close other and posttraumatic cognitions. The lack of association between social support and posttraumatic growth in the present study might be attributed to the measure of social support used (i.e., a general construct instead of comparing the influence of support from different types of interpersonal relationships, e.g., family members, partners, friends, and colleagues).

The present study also found a positive association between e-motions and e-support possibly suggesting the need for strong emotional cohesion in supportive social networks (Rimé et al., 2010). Indeed, the construct “facilitating use of e-motions” reflects not only the expression of online emotions to overcome personal difficulties but also beliefs about the usefulness of sharing and perceiving others’ e-motions in order to improve the relationships with social media contacts (Zych et al., 2017). In this view, expressing one’s own e-motions and recognizing the e-motions of other individuals might increase the likelihood of seeing new possibilities, making decisions, and perceiving e-support. The findings here described support the importance of staying virtually connected with others (e.g., by posting emotional content on social media or having someone online who shows love and affection) as a useful coping strategy in complex situations, such as the social isolation due to the COVID-19 pandemic.

The study findings also suggest that those who did not have access to offline support may use digital contexts such as social media to search for much-needed support. In support of these findings, Riva et al. (2020) discussed the potential benefits of Positive Technology to generate psychological wellbeing during the COVID-19 pandemic through three levels (hedonic; eudaimonic; social and interpersonal). It is plausible that e-motions guarantee the hedonic level (e.g., using technology to induce positive and pleasant experiences) while e-support favors the social and interpersonal level (e.g., using technology to support social integration and connectedness). Learning how to take advantage of digital technology should therefore be encouraged for individuals who are less familiar with these innovations (Bavel et al., 2020). Although social support has been identified as the stronger predictor of positive mental health, in contexts where social contact is not possible, as well as in societies characterized by geographic disconnection, it is very important to understand how communication technology can contribute in promoting posttraumatic growth and wellbeing.

However, the fact remains that much of the variance in prosocial behaviors remained unexplained and that the estimated parameters of the proposed model are small. It is possible that unconsidered factors associated with prosocial behaviors (e.g., providing social support; Kaniasty, 2020) or posttraumatic growth (e.g., rumination; Rimé et al., 2020; fear of COVID-19; Soraci et al., 2020) may also be relevant in further theoretical models. In addition, most of the literature review for the present study relied on disaster situations, such as terrorist attacks and natural disasters, which differ from the specific situation in which individuals are living in home confinement and engaging in spatial distancing (situations which are unlike the after-effects of natural disasters or terrorist attacks). Additionally, it is important to note that only 11% of the sample reported “living alone” which may have meant that the vast majority of participants in the sample was likely to have been engaging in non-online social interactions on a regular basis.

Overall, and despite the limitations of the study (e.g., cross-sectional nature of the design, self-report nature of the data, non-representative sample), the findings provide preliminary support for a digital interaction model in a novel at-risk condition that underlines how psychological and social wellbeing can be maintained despite spatial distancing. As far as the present authors are aware, the present study is the first to support a theoretical model that includes the simultaneous role of e-motions and e-support in helping individuals to cope with difficulties raised by home confinement. In addition, the proposed model underlines how positive digital interactions in times of crisis might start a virtuous cycle leading to an increase in prosocial behaviors including, for example, providing social and emotional support (both online and offline). Further studies need to validate the proposed model by using a longitudinal design with a more representative sample which would more clearly show the directionality of the effects. Based on the results, it could be hypothesized that in the initial stage of a life-threatening situation (such as a pandemic) with limited social contacts, interventions aimed at promoting use of e-motions and e-support might increase posttraumatic growth and positive mental health. This would allow society to be ready to face any challenge in the future. The successful use of digital technology by means of e-motions and e-support and the immediate availability of these resources may also serve to increase the public and governmental acceptance of such technologies for other sectors of healthcare.
